# Automating Electronic Health Record Data Quality Assessment

**DOI:** 10.1007/s10916-022-01892-2

**Published:** 2023-02-13

**Authors:** Obinwa Ozonze, Philip J. Scott, Adrian A. Hopgood

**Affiliations:** 1 School of Computing, University of Portsmouth, Buckingham Building, Lion Terrace, Portsmouth, PO1 3HE UK; 2 Institute of Management and Health, University of Wales Trinity Saint David, Lampeter, SA48 7ED UK

**Keywords:** Electronic health record (EHR), Data quality, Data quality assessment, Automation

## Abstract

Information systems such as Electronic Health Record (EHR) systems are susceptible to data quality (DQ) issues. Given the growing importance of EHR data, there is an increasing demand for strategies and tools to help ensure that available data are fit for use. However, developing reliable data quality assessment (DQA) tools necessary for guiding and evaluating improvement efforts has remained a fundamental challenge. This review examines the state of research on operationalising EHR DQA, mainly automated tooling, and highlights necessary considerations for future implementations. We reviewed 1841 articles from PubMed, Web of Science, and Scopus published between 2011 and 2021. 23 DQA programs deployed in real-world settings to assess EHR data quality (*n* = 14), and a few experimental prototypes (*n* = 9), were identified. Many of these programs investigate completeness (*n* = 15) and value conformance (*n* = 12) quality dimensions and are backed by knowledge items gathered from domain experts (*n* = 9), literature reviews and existing DQ measurements (*n* = 3). A few DQA programs also explore the feasibility of using data-driven techniques to assess EHR data quality automatically. Overall, the automation of EHR DQA is gaining traction, but current efforts are fragmented and not backed by relevant theory. Existing programs also vary in scope, type of data supported, and how measurements are sourced. There is a need to standardise programs for assessing EHR data quality, as current evidence suggests their quality may be unknown.

## Introduction

### Electronic health records (EHRs)

Electronic health record (EHR) systems play an integral role in today’s healthcare practice, enabling hospitals and other health organisations to consistently collect, organise, and provide ready access to health information. These health information systems have arguably become the standard for modern healthcare practice and are increasingly being adopted globally in many health organisations to enhance care coordination and outcomes [1–3]. They are also typified for collecting massive amounts of health data that are more reflective of the real world, with great potential for investigating a wide range of research at lower costs [4, 5]. Recent studies also show growing efforts to aggregate EHR data and, using artificial intelligence techniques, explore EHR datasets to develop models that can help improve decision-making and accelerate medical innovations and other secondary use objectives [6]. Secondary use (or reuse) here generally refers to non-direct care activities, including education, medical innovations, quality monitoring, public health surveillance, budgeting, and other commercial activities [4, 7].

### EHR data quality

The growing reuse of EHR data for secondary use can also be attributed to the expectation that it is a factual representation of patient conditions, treatment, and outcomes. These facts could be in the form of patient demographics, diagnoses, details of laboratory and pathology examinations, procedures performed, and medications ordered and administered records. Other types of documentation available in EHRs include admission and discharge summaries, lifestyle information and referral letters [3, 8]. Typically, healthcare providers capture the above record types using EHR forms and templates, scanning and speech-to-text tools [9]. Data may also be pulled into EHRs from other sources, including electronic measuring tools, clinical systems, and external data repositories.

**Fig. 1:**
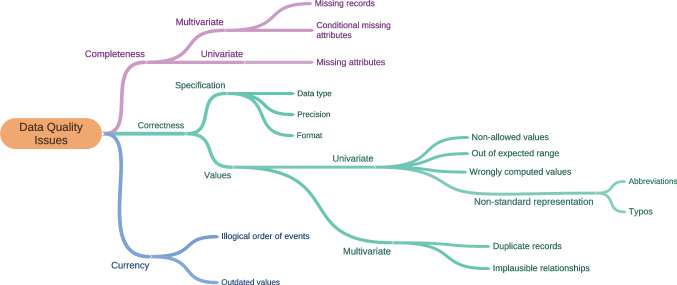
Examples of data quality problems in EHR data

Nevertheless, as with many other information systems, EHR data can be prone to variable levels of quality, particularly in terms of completeness, correctness, consistency, conformance, plausibility, and timeliness [10, 11], and are not always ready for meaningful analysis without considerable preparatory work. For example, several studies report missing timestamps and records, implausible data entries, values outside normal ranges, and duplicates. Figure 1 presents a taxonomy of commonly reported error types in EHR data. Interested readers can see [10–14].

Data quality (DQ) problems, particularly in EHRs, are the by-product of many social and technical factors, including people-related factors like work pressures, indirect data capture, misinformation from patients and other sloppy documentation practices [15–17]. Other factors such as variations in clinical practice and the lack of standardised protocols for data collection, non-intuitive EHR system design, prolonged or unsuccessful deployments, and organisational factors like workflow disruptions, staff rotations, computing aptitude and inappropriate use, such as copy and paste cultures, also inadvertently encourage the capture of low-quality health data [18–21].

Unfortunately, the cost of data quality problems is usually high, especially in industries like healthcare, negatively impacting patient safety, the quality of practice, resource management, and the credibility of clinical studies. Today, many medical errors have data errors as their root cause [22]. Data errors also affect care coordination and threaten operational efficiency, making it challenging to track programme success or respond to emerging threats [23, 24]. Equally, clinical studies and decision support tools based on EHR data also spend large sums of money on data preparation and still risk producing misleading outcomes [25–28]. There is also the consequence of an increasing volume of unusable EHR data. Given the critical impact of these DQ problems and the high propensity to reuse EHR data, measures to ensure that available EHR data are suitable and appropriate for intended use cases are essential.

### EHR data quality management

**Fig. 2:**
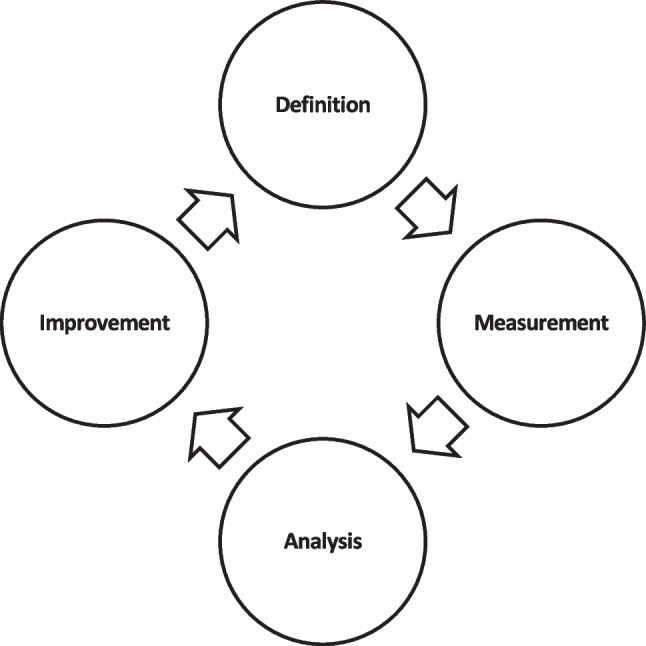
Typical DQ assessment and management framework

Prior studies note that ensuring that some given data are fit for use broadly involves four main steps: definition, measurement, analysis, and improvement activities [29–31], as shown in Fig. 2. The first step: definition, generally focuses on specifying the context of use, data elements of interest, data problem or dimensions to investigate. Measurement is the second step, and it is used to ascertain the DQ status of the dataset. Usually, this involves identifying problems in the given dataset and reporting the dataset’s status based on earlier criteria. The outcome of the measurement step is typically a collection of records with the data problems of interest and metrics depicting the degree of the identified data problems in the data sample. The third step, analysis, entails assessing the identified data problems and estimating their impact on the specified context or root causes. The measurement and analysis steps in the literature have come to be generally understood to mean assessment. The final step comprises activities to improve or make the dataset more fit for the intended use case, such as preventive and corrective procedures.

In contrast to other steps, there is a considerable amount of research on understanding and defining EHR DQ: data error dimensions, taxonomies, and quality indicators [10, 11, 32]. Several studies also present preventive interventions for improving EHR data collection and management processes. For example, some studies advocate continuous training in the use of EHR software, enforcement of standards to curb variations in documentation practice, more focus on data elements commonly needed for secondary use, giving patients more access to their data, and providing tangible incentives to encourage accurate documentation [13, 28, 33, 34]. Other studies also advocate better usability in EHR design, such as tailoring workflows to match clinical processes, and intuitive interfaces and documentation support like tooltips and input masks to guide users when in doubt and promote best practices [35, 36].

Nonetheless, assessing EHR data quality, necessary for root cause investigations, documentation training, data cleansing works, and ascertaining if implemented preventive and corrective interventions yield positive results, has remained a challenge. In many cases, data errors are rarely reported or even recognised when they occur. According to a clinical leader in one study, “…no one knows how bad data is in hospitals – on a good day, it is bad; on a bad day, it is terrible…” [37]. Meanwhile, a comprehensive data quality assessment (DQA) ensures that available EHR data are complete, consistent, and fit for use. This assessment is critical as the absence of evidence (quantitative) of the extent of the DQ problems makes creating baselines for tracking and prioritising interventions challenging [38, 39]. In addition, there are many potential benefits that EHR data consumers can derive from DQA, including improving the efficiency of data collection tools, reducing the cost of preparing EHR data for analysis, enabling clear interpretation of outcomes, and deepening the global knowledge of disease and treatments [22, 40].

### Study objectives

Several methods for assessing EHR data quality have been published in the last decade [10, 41–43]. However, many organisations implement them in an ad-hoc and manual manner, primarily via in-person audits and desk reviews that involve significant human reasoning and time, which are unsuitable for large datasets, time-constrained use cases, and tasks requiring repeated assessments [44–46]. In addition, the outcomes of these ad-hoc assessments are not readily reproducible as they are often conducted inconsistently, with assessors having varying skills and background knowledge [47, 48].

Given the high propensity for reusing EHR data, there is, therefore, a need for reliable and automated tools that can help assess EHR DQ consistently, estimate the impact of identified errors, and manage any risks involved before use. This requirement is even more crucial now, with the growing calls for improved transparency and confidence in EHR data management [10, 11, 22]. As with developing most complex systems, an explicit understanding of necessary components and their intricacies is also essential.

Hence, this review examines the state of research on EHR DQ, particularly recent approaches employed by organisations and studies to develop or implement dedicated tooling for assessing EHR DQ. Our primary goal is to identify necessary features and considerations that could guide EHR DQA tooling, not limited to dimensions and assessment methods [10, 41, 49]. This work also seeks to extend Callahan et al. [50]’s study comparing DQA approaches implemented in six US data-sharing networks. Other objectives of this review include identifying DQA programs that attempt to automate EHR DQA and the DQ problems investigated by these programs and developing a conceptual explanation of the relationships between identified features and components.

## Methods

### Search strategy and information sources

In this review, relevant articles published between February 2011 and February 2021 that discuss EHR DQA were examined using the Preferred Reporting Items for Systematic reviews and Meta-Analyses extension for Scoping Reviews (PRISMA-ScR) guideline. The articles were identified through a comprehensive search of three electronic bibliographic databases: PubMed, Web of Science, and Scopus, using the queries below:

1. (“information system” OR electronic OR computerised)
2. (medical OR health OR clinic OR hospital OR patient)
3. (“data quality” OR “data validation” OR “data integrity” OR “data error” OR “data completeness” OR “data consistency” OR “data accuracy” OR “data correctness” OR “data currency” OR “data plausibility”)
4. 2011–2021 (February 2021).

Keywords for the queries were drawn after a series of preliminary trial searches that considered the search strategies employed in related studies [10, 49, 51]. Reference lists of included papers were also checked using our eligibility criteria for articles not captured in our initial search.

### Eligibility criteria and study selection

**Fig. 3:**
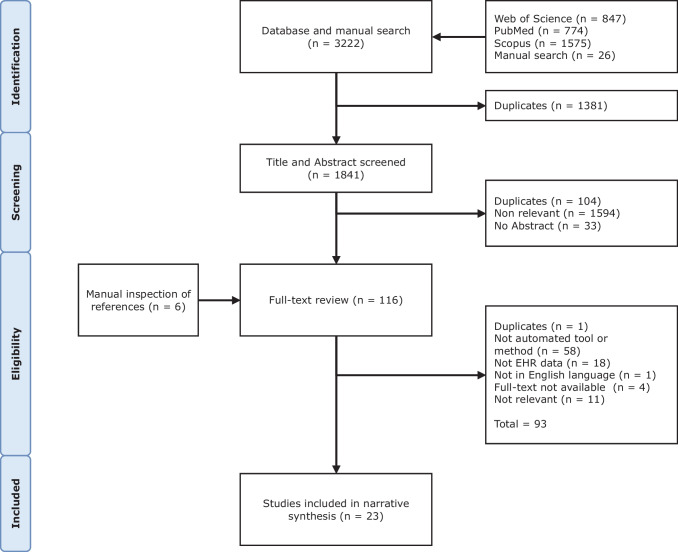
PRISMA-ScR flow diagram showing search strategy

Articles included in this review were selected based on the following criteria: (1) describe a computerised DQA program not specific to the preference of an individual user or study, (2) target data from an EHR system, and (3) be published in English. Articles that report assessments of health surveys, regional health statistics, clinical trials, and other health records not directly sourced from an EHR were excluded. One reviewer [OO] screened the titles and abstracts of 1841 articles from the literature searches and the full text of 116 relevant titles and abstracts. Of these, 26 articles were selected for a full review. [OO] and [AH] each reviewed all the 26 articles selected, while [PS] reviewed 25% (randomly selected). Disagreements were resolved by consensus, and three (*n* = 3) studies were excluded because they provided little detail about their approach or context. Figure 3 presents a flow diagram showing our search strategy and results.

### Data extraction and analysis

For each article included in this review, relevant data were abstracted using an Excel template. The data items abstracted include the author’s name, year of publication, and the name and description of the DQA program discussed. Other data items captured include the data error (DQ dimension) investigated, the context of the DQA implementation, the geographical location (country) and other design-related features and considerations. Data errors investigated were harmonised using Kahn et al. [11] definitions, cited numerous times by related studies.

Like previous related studies [10, 11, 52], we adopted an inductive and iterative approach in abstracting and codifying features and relevant considerations identified from the articles. An expanded literature review was also conducted to help refine specified features; in addition to the articles selected from the systematic search above, other articles discussing aspects relevant to developing or implementing DQA programs were reviewed, including materials such as DQ checks (rules) from large scale implementations [50, 53], DQ frameworks and published best-practices [29–31, 54–57], including those designed especially for EHR data [10, 11, 32, 43, 52, 58–63]. These additional materials were identified using Google Scholar web searches and manual searches of references in included studies.

## Results

### Study summary and context

We identified 23 articles describing dedicated DQA programs implemented between 2013 and 2021, some of which have been deployed in real-world settings to assess EHR data quality (*n* = 14) [64–77], and a few experimental prototypes (*n* = 9) [48, 78–85], as shown in Table 1. Most of the DQA programs reported are affiliated with institutions in the USA (*n* = 12) and other countries, such as the UK, Canada, Germany, Belgium, the Netherlands, and Kenya. These DQA programs were designed for various use cases, such as validating if data captured in particular EHRs conform to local system specifications [67, 72–74, 76] or if they agree with data collected in other EHRs or other health information systems [64, 75]. Also, some of the reported DQA programs focus on preparing datasets for research studies [48, 66, 79, 83] and validating that data from contributing sites conform to research network or data warehouse specifications [65, 68–71, 77]. Only a few appear generic and can be applied to different settings, data types and stages in the EHR data cycle [79, 82, 84, 85].

**Table 1:** Study summary

**Study**	**Mechanism or Tool**	**Description**	**DQ dimension** **(Kahn et al. [**11**] equivalent)**	**Year**	**Location**
Álvarez Sánchez et al. [84]	TAQIH	A web-based data exploration tool	completeness, value conformance, atemporal plausibility, uniqueness	2019	Spain
Botts et al. [64]	NIST CDA validator	Toolkit for verifying the conformance of exchanged data to health information exchange standard	value conformance	2014	US
Daymont et al. [48]	DQA toolkit	R-based toolkit for assessing paediatric growth data	atemporal plausibility	2017	US
Estiri et al. [66]	DQ^e^ -c + Vue	Toolkit for assessing completeness in a clinical data research network	completeness	2019	US
Estiri et al. [80]	DQ^e^ -c	Toolkit for assessing completeness in a clinical data repository	completeness	2018	US
Hart and Kuo [67]	Island Health DQA	Island Health Home and Community Care DQA Implementation	Defined by the user	2017	Canada
Huser et al. [68]	ACHILLES Heel	An open-source software that provides a useful starter set of rules for preparing data for use in a CDRN	value conformance, plausibility	2016	US
Johnson et al. [79]	DQA toolkit	Python implementation of the HDQF framework	completeness, atemporal plausibility, value conformance	2019	US
Juárez et al. [69]	QR generator	A toolkit for validating data in local data warehouses in a distributed research network	Defined by the user	2019	Germany
Kapsner et al. [70]	DQA Toolkit (R)	A toolkit for preparing data for use in a research network	conformance, completeness and plausibility	2019	Germany
Khare et al. [71]	PEDSnet Data Quality	Software implementation of the DQA program at PEDSnet CDRN	completeness, plausibility, value conformance, relational conformance	2019	US
Lack et al. [72]	DQA toolkit (C++)	Software implementation of a DQA program for detecting errors in treatment planning workflows at a health facility	conformance, completeness and plausibility	2018	US
Monda et al. [73]	Extended OpenMRS	DQA module implemented within an openMRS EHR software	Defined by the user	2013	Kenya
Nasir et al. [81]	DCAP	A tool for determining the completeness of individual patient records	completeness	2016	US
Noselli et al. [85]	MonAT	A web-based data exploration tool	completeness, plausibility, value conformance	2017	UK
Qualls et al. [65]	Self-contained package	A package containing DQ analysis programs for network partners within the PCORnet DRN	conformance, completeness and plausibility	2018	US
Rabia et al. [74]	DQA Toolkit	Rule-based implementation of a DQA program for assessing discharge summaries	completeness, atemporal plausibility	2018	Algeria
Ranade-Kharkar et al. [75]	HIE Data Adjudicator	Toolkit for assessing the quality of data entering or leaving a health information exchange framework	plausibility, completeness	2014	US
Silva et al. [82]	DICOM Validator	A web service for validating the conformance of EHR data produced by PACS to DICOM standards	value conformance	2019	Portugal
Tute et al. [78]	openCQA	A DQA tool that uses openEHR specifications to enable interoperable assessments	Defined by the user	2021	Germany
van der Bij et al. [76]	DQ Feedback tool	A feedback tool that evaluates differences in EHR data among practices and software packages	conformance, completeness	2017	Netherlands
Vanbrabant et al. [83]	DAQAPO-package	A toolkit based on R that enables automated assessment of EHR data for emergency department simulations	completeness, temporal plausibility, atemporal plausibility, uniqueness	2019	Belgium
Walker et al. [77]	QA program (‘emrAdapter’)	A toolkit for validating data in local data warehouses in the CER research network	value conformance, plausibility, completeness	2014	US

### Design features and considerations

**Fig. 4:**
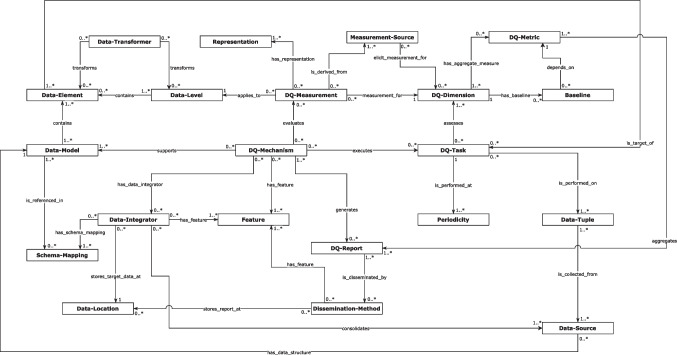
UML representation of concepts for operationalising EHR data quality assessments

We identified 24 features and considerations necessary for operationalising EHR DQA. These features have been grouped under five top-level categories that include: defining DQA tasks (*DQ-Task*), acquiring and managing measurements (decision-making criteria) and other computational resources used to evaluate defined DQA tasks (*DQ-Measurement*), collecting and managing target data (*Target-Data*), mechanisms for implementing measures (*DQ-Mechanisms*), and disseminating outcomes (*DQ-Report*) as shown in Table 2. We describe these categories and their interrelationships in Fig. 4 and in the following subsections.

**Table 2:** Mapping of EHR DQA programs to concepts identified

**SN**	**Main category**	**Low-level concepts**	**Description**	**Example instances**
**1**	DQ-Task	DQ-Task	Specifications for the DQA activity	
**2**	DQ-Task	DQ-Dimension	Data error to investigate, quality properties determining how well data are fit for use, or label for grouping measurements	Completeness [64–66, 69, 71, 74, 75, 77, 79–81, 83, 84], conformance,[64, 65, 68, 70, 71, 77, 82] plausibility [48, 65, 70, 71, 73, 77, 83], consistency [74, 75, 79, 83], accuracy [69, 84], timeliness [75], out of range [73, 83], representation completeness [78, 79], domain completeness [78, 79], domain constraints [78, 79], syntax accuracy [69], duplicate [83], domain consistency [79], precision [74], violations of logical order [83], redundancy [84], readability [84].
**3**	DQ-Task	Data-Element	An individual unit of an observation	Data elements determined at runtime [73, 78, 81, 84] pre-defined data elements, e.g. growth measurements [48], discharge summaries [74], and emergency records [83].
**4**	DQ-Task	DQ-Metric	An aggregate measure for assessing defined *DQ-Dimensions*	Simple ratio [69, 73, 75, 79, 80], counts [66, 70, 73, 77], weighted scores [81, 84], and Boolean values[73].
**5**	DQ-Task	Baseline	A threshold for judging the level of a DQ-Dimension in a dataset for a particular use case	Greater than 99.9% [67] and 90%[79], user-defined [73, 84], previous *DQ-Metric* score [65, 74].
**6**	DQ-Task	Periodicity	The type of execution and frequency supported	On-demand [68, 81, 83] scheduled e.g. every 24 h [72], quarter [66] and other specified intervals [65, 67, 71].
**7**	DQ-Task	Application area	The point in the EHR data cycle where the DQA program or tool would be applicable	Directly on EHRs data stores [72, 74], EHR data exchanged via health information exchange frameworks [64, 75]
**8**	DQ-Task	Priority	The rationale for focusing on selected dimensions and data elements	Data elements type supported by available measurement [71, 84], data elements are necessary for intended use cases [71, 72], dimensions prevalent in previous records and literature [65, 84], dimensions for which measurements and required data are available [65], demands of internal and external data consumers [71].
**9**	Target-Data	Target-Data	One or more tuples containing observations	
**10**	Target-Data	Data-Source	The range of sources or datasets that the program can be applied to	Single [72, 74, 75], multiple[68–70, 78]
**11**	Target-Data	Data connection	The method for accessing data sources. DQA program can support more than one type of connection	CSV files [70, 84], database scripts or connections [70–72, 74, 80, 81], REST API [69], Health Level Seven (HL7) document [75], XML[77].
**12**	Target-Data	Data-Integrator	The method for consolidating data from different sources into a single model or view.	Extract, transform and load (ETL) [68, 69, 71, 76, 77]
**13**	Target-Data	Data-Model	Logical representation of data elements, their relationships, and constraints that is used to enable other components to operate and share the same data uniformly	Observational Medical Outcomes Partnership (OMOP) [68, 69, 80], extended OMOP [71], Clinical Research Document [77], openEHR,[78], PCORnet [65, 66, 80] Informatics for Integrating Biology & the Bedside (i2b2) [70], Digital Imaging and Communications in Medicine (DICOM) [72, 82], National Summary Care Record Format [76], locally defined standards [69, 81].
**14**	Target-Data	Data-Location	The physical location of the Target-Data	Users’ repository [68, 69, 77], central server [71, 76]
**15**	Target-Data	Size	The amount of data the program can support or has been validated with.	Small (0-100k) [74, 77], medium (100k to 1 M) [79, 80], large (1 M+) [68].
**16**	Target-Data	Data-Transformer	Functions for converting data from one format, structure and value to another	Vocabulary crosswalks [71, 75]
**17**	DQ-Measurement	DQ-Measurement	Criteria for measuring DQ-Dimension	
**18**	DQ-Measurement	Data-Level	This refers to the data level considered in the DQ measurement.	Cell level [69], field level [65, 67, 70, 84], record level [74, 81, 83], table level [65, 67, 71].
**19**	DQ-Measurement	Measurement-Source	Method for creating measurements and accompanying reference items	Domain experts [68–72, 79, 80], crowdsourcing [68, 71], data standards or dictionaries [71, 77, 78], national guidelines [76], literature review [71], statistical analysis [83, 84].
**20**	DQ-Measurement	Representation	Format for representing measurements	Natural text [68, 72, 80], conditional logic statements [75, 78, 79], database queries [67, 69, 70, 73, 78], metadata repository[67, 69], programming language scripts [71, 73, 83], mathematical and computational models [48, 74, 81].
**21**	DQ-Report	DQ-Report	The content of reports and type of analysis	Summary metrics [69], DQA metadata [67, 79], date and time the result was obtained [67, 71], severity warnings or comments [64, 65, 68, 82], error message to display [68, 71, 73], data profile of source data [68, 80], records returned per dataset or site [77], records returned linked to assessment metadata [67, 69, 70, 72, 73, 83, 84], aggregate results from multiple assessments or sites [66, 70, 77], results grouped by data element [66–68, 71, 83], suggestions on improvements [64], information to exclude [69].
**22**	DQ-Report	Dissemination-Method	Techniques or tools for communicating assessment methods	Store results in a repository [66, 67, 69, 70, 80], file export [71, 76, 77], Tables [68, 70, 73], charts [66, 68, 79, 80, 84], longitudinal views [66], collaborative workspace, e.g. Github [71]
**23**	DQ-Mechanism	DQ-Mechanism	The mechanism for operationalising DQA components	Visualisation tool [84], dedicated tool [48, 68, 71, 80, 83]
**24**	DQ-Mechanism	Feature	Functions that enable a DQ-Mechanism to perform satisfactorily and meet Stakeholder requirements	See Table 3.

#### DQ-task

This category describes the specifications for the DQA activity, which may be defined formally or informally by *Stakeholders*, internal or external, to the individual or organisation conducting the DQA activity, such as data consumers, program developers, data producers and host institutions [30]. Typical items in a *DQ-Task* include specifications directly related to quality, such as the dimensions to investigate (*DQ-Dimension*), the data elements of interest, and a metric or baseline for ascertaining whether a dataset is good enough for the intended use case (*DQ-Metric*). *DQ-Task* specification may also include non-functional specifications such as how it should be conducted, such as the *Periodicity* at which a DQA activity should be conducted, such as if it should be conducted on-demand [68, 83], autonomously or at set intervals, e.g., at the end of every day [65, 72].

Generally, a *DQ-Task* aims to assess one or more *DQ-Dimensions* in a given dataset, which could be a measurable quality property, a collection of related measurements, or database queries such as those used for many retrospective analyses like case identification [10, 11, 42]. As observed in this study, the definitions of these *DQ-Dimensions* often vary and are derived from disparate sources, including domain experts, literature reviews, and previous records of errors [65, 71, 72]. Some national bodies and research communities also prescribe *DQ-Dimension* definitions for specific intended use cases, like intervention monitoring and aggregating data into federated networks [65, 70, 76, 77, 86]. Also, given the increasingly task-dependent requirement of many DQA activities, some programs allow data consumers to specify the *DQ-Dimensions* they want to investigate dynamically at runtime [69, 73]. These definitions can be expressed in different formats, including natural language (text), ontologies [52], taxonomies [39, 74, 83], queries [77, 87], and other computational structures. Terms used to describe *DQ-Dimension* may also have multiple connotations. For example, completeness is a typical *DQ-Dimension* instance. The term has been used to describe records with missing values, values not in the desired formats, or data elements with insufficient information (predictive value) for the intended use [80, 88]. DQA programs with the additional requirement of comparing outcomes, root cause analysis, and implementing improvements might find this ambiguity property problematic.

Selecting the *DQ-Dimensions* to assess is another critical consideration in defining *DQ-Tasks* as it indicates the coverage of the DQA activity and the type of measurements (*DQ-Measurement*) that will be required. In some instances, the *DQ-Dimension* selected may also determine targetable data elements and levels in a DQA activity because certain *DQ-Measurements* may only be applicable for data elements of a particular domain, data type, and level [56, 65, 89]. Similarly, it is unlikely that DQA programs will be able to evaluate all possible *DQ-Dimensions* against all available data elements, especially for large EHR datasets, which often have diverse stakeholders. Some required *DQ-Measurements* may be unavailable or too complicated to operationalise [49, 90]. Equally, datasets with many attributes, complex data types, such as images, and large sizes, could demand more resources beyond the mechanism (*DQ-Mechanism*) available to execute the *DQ-Task*. So, for such scenarios, trade-offs between *DQ-Dimensions*, data elements, time, and capability of the *DQ-Mechanism* are essential to improving the efficiency of the DQA activity. Examples of such trade-offs could include focusing on data elements necessary for intended use cases [72, 76, 91], those prevalent in the previous records [61] and literature reviews [61, 84], or having more weight regarding their contributions to the overall quality of a dataset [92]. A *DQ-Task* may also be limited to *DQ-Dimensions* that are feasible to investigate, i.e., required measurements and data are available [61, 65] or data elements with a high return on investment (the tendency of finding data elements in most datasets) [88]. In this study, we have used the term *Priority* to represent such trade-offs and their rationale. Capturing this information is essential for transparency. It also helps to ensure that organisations’ DQA coverage expands progressively.

Furthermore, depending on the intended goal, a *DQ-Task* may include a metric and a baseline for determining if the target dataset is good enough for the intended use case. This metric (*DQ-Metric*), which is an aggregate score, could be quantitative (e,g., count [66, 77], simple ratio [73, 80], percentage), categorical (e.g., ordinal, Boolean [73]) or other complex metrics [54, 93]. As inferred from this review, these metrics are applied to aggregated outcomes of *DQ-Measurements* across different data levels (field, record, table). They help present assessment results in easily digestible and comparable formats [13, 68] and may be embedded as part of *DQ-Measurements* given their close associations.

#### DQ-measurement

This category refers to the criteria for evaluating selected *DQ-Dimensions*. It typically encompasses one or more comparisons involving data elements’ content, derivation, property (e.g., type, format) and reference items across different data levels (cell, record, table). In this review, target data elements are subsets of the data elements defined in the *DQ-Task* definition and the data model. A data model is described in the next section. Reference items can be any values held in other data elements in the same dataset, the outcome of other *DQ-Measurements* and explicitly defined values, like numbers, Boolean, text, value ranges, regular expression, and value sets [10]. The data type of the data element evaluated may determine the kind of reference item required. For example, range and spelling checks would likely be used to assess data elements of type numeric and text.

Common comparisons include assessing value conformance, such as values presence, conformance to defined patterns, precision, allowable ranges or value sets, functional dependencies and causal relationships [10, 11]. It may also involve evaluating agreement with other data sources like a previous snapshot of the same data, other datasets within the same or different EHR systems, and recollected observations [94, 95]. For *DQ-Measurements* involving disparate datasets, it is essential to note that the datasets may have syntactical and semantical differences. And while various transformation functions and tools exist to normalise datasets, excessive transformations can overestimate or underestimate *DQ-Dimensions*.

Furthermore, as stated earlier, *DQ-Measurements* apply to specific data levels (cell, field, record, table) [11, 43, 50, 56, 89]. For instance, in assessing value conformance, *DQ-Measurements* may target single data cells in records, such as checking if single data cells match specifications like data type and format [67, 88, 89]. In the same way, some *DQ-Measurements* apply to the field level, comparing the output of aggregating selected observations (records) in that field with reference information, such as identifying univariate outliers and evaluating redundancy [43, 68]. Others involve multiple data elements across a record level, such as identifying functional dependency violations [39, 83] and agreement between multiple variables like fields containing diagnoses and medication concepts [50, 53]. Likewise, multiple data elements can also be compared across aggregated records, such as comparing the value of a data element with successive values of the same fields for a given subject to determine if values changed implausibly over time. It is also possible for *DQ-Measurements* to act on the table level and for multiple *DQ-Measurements* to be combined using logical junctions like AND, OR, and NOT to investigate complex *DQ-Dimensions* [67].

Like *DQ-Dimensions*, the logic for *DQ-Measurements* may be acquired from multiple knowledge sources, including domain experts [68–72, 79, 80], data consumers [69, 73], crowdsourcing [53, 68, 71], data standards or dictionaries [71, 77, 78], national guidelines [44], literature review [71], and other existing *DQ-Measurements* [53]. Studies have also shown that it is possible to create *DQ-Measurement*s inductively from datasets using statistical measures, natural language processing (NLP), machine learning and rule mining techniques, which also offer automated capabilities [96–100]. Nevertheless, acquiring *DQ-Measurements* from these sources may involve varying confidence, coverage, and acquisitional efficiency. For instance, domain experts can produce *DQ-Measurements* via interviews and crowdsourcing, which may command high confidence locally, but could also be expensive, time-consuming and have low coverage [97, 101]. Likewise, *DQ-Measurements* developed using data-driven techniques can be inconsistent, unexplainable, and prone to false positives.

#### Target-data

This category encompasses considerations in handling input data in a DQA activity, including how it will be accessed, supported formats, and data storage. Some methods reportedly used for accessing EHR data for assessment include direct execution of database scripts and accessing health information exchange frameworks like openEHR [78]. *Target-Data* have also been extracted from EHR repositories and made available in filesystem formats like comma-separated-values (CSV) [84, 87]. The approach employed to access EHR data is often determined by host environments, data protection policies, infrastructure, performance, and interconnectivity. For instance, some institutions require EHR data to be accessed remotely to enable more autonomy over their data and reduce the likelihood of security and privacy breaches [77, 78]. Size is another factor, as it is not always timely, economical, or safe to inspect every record in a given data source [58, 63, 68, 79]. So, instead of assessing the whole dataset, subsets of the original data may be selected using sampling and randomisation strategies [40, 58]. However, assessment outcomes do not always reflect the dataset’s DQ status. Also, determining the appropriate dataset size sufficient to estimate the state of the whole dataset can be challenging [58].

Furthermore, a *DQ-Task* could also entail comparing or assessing *Target-Data* that use different syntactical and semantical standards to store data. To help ensure all components operate and share data uniformly, some DQA programs employ Common Data Models (CDMs). Examples of commonly reported CDMs include the Observational Medical Outcomes Partnership (OMOP) CDM [68, 71, 80], Sentinel CDM (SCDM) [50], Informatics for Integrating Biology & the Bedside (i2b2) [70], Digital Imaging and Communications in Medicine (DICOM) [72, 82], and openEHR [78]. These CDMS contain varying data elements defined for a particular aggregated form, institution, or use case [68, 70, 78, 80] and linked differently [60, 77, 102]. In most instances, only a single CDM is supported, which is, apparently, more straightforward to implement. However, this approach limits DQA programs and makes them not generalisable and scalable to other sites [80, 103]. With more institutions exchanging and aggregating data, there would likely be more demand for DQA programs to support multiple data structures and study designs.

Similarly, EHR data are not always in the same structure as the specified CDM. In such scenarios, data integration is required. Common approaches for integrating data sources include extraction transformation and loading (ETL) activities, data replication, or a virtual representation [104]. These data integration activities often require pre-defined schema mappings of source and target data models, which can be hardcoded, or defined dynamically using interactive interfaces, configuration files and other semi- or fully automated approaches [60, 104]. In addition, data transformation may be required to convert source data, especially unstructured data, to a format appropriate for target *DQ-Measurements* [29, 105].

#### DQ-report

This component refers to the content and verbosity of the outcomes from executing a *DQ-Task*. It provides feedback to enable stakeholders to judge their datasets, including remediation recommendations, which can trigger and shape improvement efforts. For instance, a typical *DQ-Report* content may contain a collection of returned records that satisfy the *DQ-Dimensions* evaluated, *DQ-Metric* scores and metadata containing details of other concepts involved in the DQA process, including possible enhancements. These outcomes can be communicated to *Stakeholders* using a preferred *Dissemination-Method* like tables and graphs that allow for quick analysis and provide visual attributes for drawing attention to specific results and details. *DQ-Report* can also be exported to relevant bodies or stored for further analysis. Similarly, *Dissemination-Methods* may also incorporate f*eatures* that enable them to fulfil reporting requirements, such as interface designs, password protection, anonymisation functions and secured data transfers, as discussed below.

#### DQ-mechanism

This category refers to the program, process or tools employed to operationalise the different activities involved in executing a *DQ-Task* and the features that enable them to perform satisfactorily and meet stakeholders’ requirements. Commonly reported features identified in this review have been grouped under configurability, usability, scalability, performance, and security, as shown in Table 3 below.

**Table 3:** Example of *DQ-Mechanism* features

**Feature**	**Description**	**Examples**
**Configurability**	Allow users to personalise, adapt or extend the DQA process to match their requirements or environment	*DQ-Dimension* [73, 78] Weights in *DQ-Metric* [81, 84] *Baselines* [84] *DQ-Measurement* [69, 73] *Schema-Mapping* [69, 80, 81]
**Usability**	Enable users to perform tasks efficiently and effectively	Graphic user interface [66, 84] Interactive options [66]
**Scalability**	Enable the system to sustainably respond to changes in resource demand, datasets, or environment	Modular design [80] Multi *Data-Model* support [80] Interoperable *DQ-Measurement* [78] Fast deployment [70]
**Performance**	Enable the system to maintain satisfactory levels of responsiveness and stability for specified workloads	Parallel computing Out-of-memory execution [80] Batch processing [77, 82] Sampling strategy [58, 77]
**Security**	Ensure security concerns such as privacy and proprietary protections are satisfied	Password protection [66, 80] Secured file sharing [66] De-identification [72, 76, 82]

## Discussion

This review examines recent efforts to automate EHR DQA. So far, we have identified 23 DQA programs, with more than 80% implemented within the last five years (at the time of the search). This trend shows organisations using EHR data for analysis are becoming more aware of the inherent quality problems. It also affirms the growing focus on automating EHR DQA, driven mainly by the need to help researchers prepare EHR data to meet research objectives. However, only a few DQA programs currently focus on improving the data quality at source EHRs, which is critical for preventing immediate medical and operational mishaps and improving electronic documentation.

The latter can be attributed to available DQA programs not being as robust as desired, focusing on DQ dimensions, such as completeness and value conformance, which can be considered trivial to implement and are currently being supported by various data integration and analytic tools. Also, unstructured data formats like free text and images, which make up most data stored in EHRs [7], are computationally more challenging to analyse [8] and hence rarely supported. Similarly, many of the reported DQA programs are tightly coupled to existing infrastructure and are available only to users of the same community. Some of them are also too technical, lack interactivity and require users to know about the host systems and supported programming languages, like knowledge of R, to operate the DQA tool [66, 68]. They are also not being evaluated adequately; hence, they are not ready for general clinical use.

These limitations further emphasise the challenge of conducting EHR DQA. Interestingly, our extended review showed no lack of frameworks discussing DQ theories, best practices, and other concepts associated with DQA. For instance, several frameworks like the Total Data Quality Management (TDQM) framework describe best practices for improving overall DQ and conducting DQA from a general perspective [29, 30, 54–57] and a few others tailored explicitly for EHR data [10, 11, 32, 52]. However, it is unclear how the many theoretical concepts can be translated into practice, amongst other factors. For example, many existing frameworks focus on standardising DQ dimensions and identifying potential assessment methods, but they do not provide much regarding how these methods can be operationalised in real-world settings. Also, only a handful of studies investigate other critical aspects of DQA, such as data management [43, 58, 60] and reporting and applying outcomes [59, 63]. The concepts are also discussed in isolation and, thus, contain competing and ambiguous terms, which introduce confusion and make it difficult to translate them into practice [38, 80].

### Strengths and limitations

This study identifies several programs and tools developed, implemented, or adopted for automating EHR DQA using a systematic approach. In addition to previous studies using this approach, our choice was also motivated by the benefits of not limiting our analysis to the authors’ preconceptions and the ability to organise information and assumptions explicitly. However, the list of DQA programs identified may not be exhaustive as we focused on only those published in selected bibliographic databases. Unpublished programs or those available to select users, including proprietary programs, were outside the scope of this review.

Nonetheless, this review identified several critical components and considerations in developing and operationalising DQA programs for EHR data. These components have been grouped under five top-level categories: defining DQ tasks, developing and managing measurements for inspecting datasets, collecting and handling target datasets for assessment, analysing and disseminating outcomes, and mechanisms for operationalising all these components. As shown in Fig. 4, we have explained these categories extensively using UML diagram concepts and domain-independent terms derived from standard ontologies, like the Basic Formal Ontology [106] and other reviewed frameworks, in our attempt to disambiguate the so-called complex activity of conducting EHR DQA. The components identified have also been organised to reflect expected knowledge requirements and practicality. This is intended to foster better collaboration between stakeholders, such as data owners, reporting teams, and knowledge curators, and encourage the reuse of resources like data integration tools, rule engines, and reporting frameworks. It also allows each component to be standardised individually against having one general standard. Furthermore, we anticipate that the identified concepts can help to curate knowledge of the different approaches to DQA, which is a bold step toward standardising health data quality assessment, as demonstrated in Table 2.

This work has some similarities with existing works and some essential additions, even though expressed in different languages in some cases. For instance, it recognises the task-dependent nature of DQA and the importance of a well-defined plan [50, 52]. In addition to specifying DQ dimensions to assess, it notes that how assessments are conducted shapes the scope and contributes to the variability of DQA processes, such as the periodicity of checks and prioritisation strategy. Similarly, while there is no unique way of measuring DQ dimensions, this review explicitly expounds on the structure and complexities involved in developing and managing DQ measurements, which could help reduce the confusion surrounding the development of new assessment methods. In addition, this works attempts to propose a relationship between DQ concepts and attributes, which have been mentioned in isolation in various existing works, as shown in Fig. 4.

Nonetheless, this review has a task-centric focus, emphasising technological-related components reported in the literature. Also, while we took great care to ensure that the literature search was broad and systematic, our findings may be missing some necessary components not discussed in the articles reviewed. This study did not also elicit the views of the different EHR data users to validate the findings from this review. So, while our results reflect shared conceptualisations across the literature and considerations that could apply uniformly, further research may benefit from more validation, including obtaining stakeholder input on the utility of our contribution in practice.

## Conclusion

EHR data are a critical component of today’s healthcare industry and must be good enough to support clinical care or other secondary use cases. Various strategies have been proposed to ensure this, including DQA activities for detecting problems that need attention. Nevertheless, anecdotal evidence suggests an absence of comprehensive tools for facilitating reliable and consistent assessments. In light of this, we have examined the literature in this study to assess this gap and identify important considerations for developing and implementing new DQA tools. Our findings show that automating EHR DQA is gaining traction. However, there appears to be a general lack of clarity surrounding DQA processes brought about by the contextual nature of DQ requirements, heterogeneity of EHR data, and the challenge of developing measurements for inspecting datasets. More worrisome is that the quality of these processes is unknown as, in many cases, they are not backed by theoretical frameworks, and there are no obligations to certify that DQA tools measure what they are designed to measure. There is also a growing demand for interoperable checks that apply to multiple contexts. Healthcare organisations hoping to develop DQA programs will find this review helpful as we have summarised what exists and shed light on critical components required to operationalise DQA processes. We also anticipate that this work would help reduce the confusion around EHR data quality management and provide guidance appropriate for developing effective programs.

## Data Availability

The datasets generated and analysed during the current study are available from the corresponding author on reasonable request.
